# A Fast Prediction Method for Wide-Angle Bistatic Scattering and Reflection Coefficients of Acoustically Coated Plates

**DOI:** 10.3390/s26061899

**Published:** 2026-03-18

**Authors:** Yanhua Zhang, Zilong Peng, Liwen Tan, Shihao Wu, Enze Lv

**Affiliations:** School of Energy and Power Engineering, Jiangsu University of Science and Technology, Zhenjiang 212100, China; 231110803112@stu.just.edu.cn (Y.Z.); tlwsjtu@gmail.com (L.T.); 231110803107@stu.just.edu.cn (S.W.); 231210803108@stu.just.edu.cn (E.L.)

**Keywords:** bistatic scattering, bistatic reflection coefficient, acoustically coated plate, scattering prediction

## Abstract

Multistatic sonar provides enhanced target detection in complex underwater environments. The wide-angle bistatic scattering characteristics of targets, particularly the bistatic reflection coefficients, are important for evaluating system performance and designing acoustic absorbing coatings. However, obtaining full-angle experimental measurements is challenging, and conventional finite-element simulations become computationally prohibitive for large structures, high frequencies, or exhaustive angle sweeps. To overcome these challenges, a fast wide-angle scattering prediction method for acoustically coated plates is proposed. The method constructs a scattering transfer matrix from the surface mesh and retrieves the equivalent source density from a small subset of scattered-pressure samples, enabling reconstruction of the full-angle scattering field and rapid extraction of reflection coefficients. The approach is demonstrated on both rigid and coated plates, with predictions compared against finite-element calculations. The results demonstrate that the proposed method accurately reproduces the bistatic reflection coefficients, including non-linear dispersion effects and interference fringes, across a wide frequency band from 100 Hz to 5 kHz. Compared to traditional FEM sweeps, this method significantly reduces computational time while maintaining high accuracy, providing an efficient tool for the design of acoustic stealth materials and laying a foundation for rapid target strength prediction of complex targets using the Planar Element Method.

## 1. Introduction

The scattered acoustic field of underwater targets is a critical factor in active sonar detection, target classification, and acoustic stealth design [1,2]. Analytical solutions exist for simple geometries, such as spheres [3,4] and infinitely long cylinders [5], using separation-of-variables techniques. However, targets with arbitrary shapes typically require numerical methods, including the T-matrix [6], finite element/boundary element (FEM/BEM) [7], or finite-difference time-domain (FDTD) approaches [8]. As target size, frequency, and angular coverage increase, these methods become computationally expensive, limiting practical applicability. To address the limitations of traditional numerical methods in high-frequency acoustic scattering, Ihlenburg et al. [9] investigated the pollution error in finite element solutions for time-harmonic wave problems, demonstrating its dependence on wave number, mesh design, and material properties in fluid–solid interactions. Furthermore, Redonnet and Cunha [10] proposed a hybrid approach that effectively reduces computational cost by combining near-field acoustic holography with computational aeroacoustics, thereby enabling efficient prediction of the radiated sound field from complex sources. These studies highlight that, although high-fidelity numerical methods can provide accurate results, their efficiency remains a major challenge for large-scale and high-frequency scattering problems.

To address high-frequency scattering efficiently, physical acoustics approaches have been widely adopted. The Kirchhoff approximation provides a foundational framework, while the facet-based method proposed by Fan et al. [11,12] and Zheng et al. [13] approximates curved surfaces using locally planar elements, enabling efficient modeling of complex targets. Further accuracy improvements were achieved by Sammelmann [14] and Xue et al. [15] through Gaussian–Legendre integration over rectangular and curved triangular elements. For scenarios involving shadowing or multiple scattering paths, Chai et al. [16] introduced a ray-tracing-based fast prediction method. Despite their efficiency, these ray-based methods often fail to fully capture the complex wave phenomena associated with surface elasticity and diffraction, particularly when viscoelastic coatings are involved.

Multistatic sonar systems, offering enhanced angular diversity and interference resilience, are increasingly applied in complex environments [17,18,19]. Nevertheless, in bistatic or multistatic configurations, reflection coefficients exhibit strong angular coupling. Obtaining a complete bistatic scattering map requires an exhaustive traversal of incident–receiving angle combinations, which incurs a prohibitive computational burden for conventional FEM simulations.

In underwater acoustic stealth, high-loss viscoelastic coatings are widely applied to absorb incident sound and reduce echoes. These coatings often contain internal structural units, and their performance is influenced by material properties, dispersion, interfacial dissipation, and structural scale. For example, Gao et al. [20] enhanced low-frequency absorption by introducing conical air cavities, while Wang et al. [21] achieved multi-band absorption using periodic steel scatterers. Ranjbar et al. [22] systematically investigated coatings incorporating various cavity geometries. However, efficiently predicting the wide-angle bistatic reflection coefficients of these finite coated structures—without relying on time-consuming full-wave sweeps—remains an unresolved issue in the existing literature. While T-matrix methods have been successfully applied by Waterman [23] to bounded obstacles, Boundary Element Methods (BEM) have been optimized by Koopmann and Benner [24], time-domain extensions by Lee et al. [25], reflection-coefficient studies in underwater sensor networks by Sathish et al. [26], and active-control strategies by Timmermann et al. [27], these approaches often struggle with the heavy computational load required for wide-band, wide-angle sweeps of coated structures.

To reduce computational complexity, Schenck et al. [28] proposed a transfer-matrix-based equivalent source density representation, enabling reconstruction of full-space scattering fields from limited data. This concept was subsequently extended by Chen et al. [29,30] and Gu et al. [31] to spheres, multilayer stiffened cylindrical shells, and complex underwater vehicles, achieving accurate scattering predictions using only a small number of scattered pressure measurements. Yet these studies primarily focused on rigid bodies or simplified shells. The application of such fast prediction methods specifically to finite plates with high-loss acoustic coating for retrieving broadband bistatic reflection coefficients has not been sufficiently characterized.

To address the inherent trade-off between computational efficiency and physical fidelity in existing methods, this paper proposes a fast wide-angle scattering prediction method for finite-sized coated plates based on the acoustic transfer matrix and equivalent source density inversion. While this approach may not constitute the discovery of new physical laws, it represents a substantial and significant applied engineering innovation built upon a novel combination of established tools. Specifically, by constructing the acoustic transfer function of the surface mesh and directly integrating the reconstructed full-angle scattering field with the bistatic reflection coefficient formula, the method achieves rapid extraction of wide-angle reflection characteristics using only a very small number of monostatic and bistatic scattered pressure samples. Compared with the existing literature, the main innovative contributions of this study are as follows:

(1) Unlike full-angle sweeps using the Finite Element Method (FEM), which demand enormous computational resources, the proposed method can reconstruct the full-space bistatic scattering field using only a sparse subset of monostatic and bistatic samples, while preserving the high- fidelity of numerical simulations and significantly reducing computational time.
(2) In contrast to ray-tracing or physical acoustics approximations that simplify surface interactions, the proposed method can accurately reproduce the nonlinear dispersion effects and high-frequency interference fringes induced by viscoelastic coatings, effectively bridging the gap between computational efficiency and physical accuracy in the analysis of finite-sized acoustic coating structures.
(3) The validity of the method is extended to the frequency range of 100 Hz–5 kHz, verifying its robust prediction capability from the low-frequency regime dominated by diffraction to the high-frequency regime dominated by interference. This provides a comprehensive tool for the rapid design and evaluation of acoustic stealth materials within the operating band of active sonar.
(4) Furthermore, the method is engineered for direct integration with the Planar Element Method (PEM) and Kirchhoff approximations. The obtained wide-angle bistatic reflection coefficients can be used as localized inputs, enabling rapid target-strength prediction of finite-sized acoustically coated structures at higher frequencies while bypassing the computational burden of full-wave FEM for large-scale underwater targets.

The remainder of this paper is organized as follows: Section 2 details the theoretical formulation of the bistatic scattering transformation and the step-by-step numerical implementation of the proposed fast prediction method. Section 3 presents the validation results, comparing the predicted scattering fields and reflection coefficients against full-wave FEM simulations for both rigid and coated plates, including a detailed analysis of computational efficiency and error. Finally, Section 4 summarizes the conclusions and discusses future applications.

## 2. Methods

### 2.1. Principle of Bistatic Scattering Transformation

**Figure 1:**
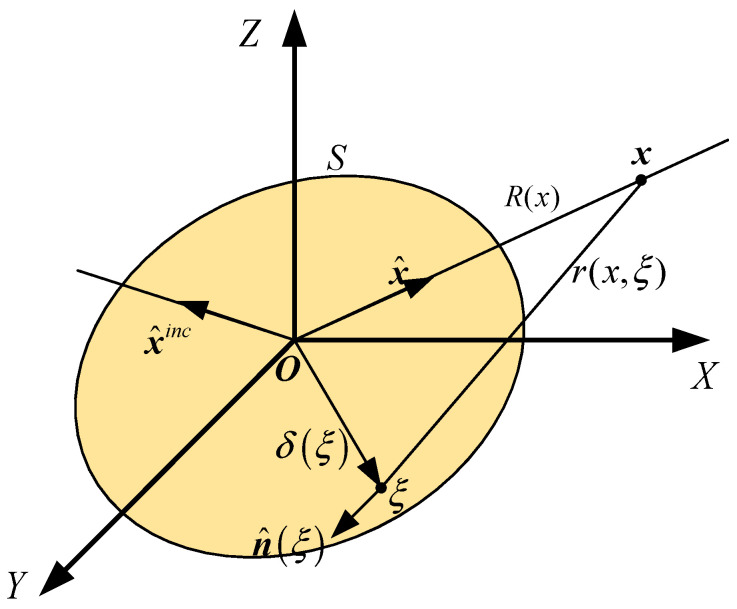
Schematic diagram of target acoustic scattering.

The schematic diagram of target acoustic scattering is shown in Figure 1. The scattered acoustic field can be expressed as the product of the acoustic transfer function and the equivalent source density function on the target surface [28]. Under plane-wave incidence from a given direction ${\hat{x}}^{inc}$ , the resulting pressure at a receiving point x can be written as:

(1) $$p^{S}(x,{\hat{x}}^{inc})=\frac{1}{4\pi }\int_{S}q(\xi ,{\hat{x}}^{inc})\left[\frac{\partial }{\partial n_{\xi }}+i\right]\frac{e^{-ikr\left(x,\xi \right)}}{r\left(x,\xi \right)}dS(\xi ),$$

here,

-
x denotes the receiver position;
-
${\hat{x}}^{inc}$ indicates the direction of the incident wave;
-
S represents the target surface;
-
q denotes the unknown sound source density function;
-
ξ corresponds to a point on the target surface;
-
r(x,ξ) is the distance between the target surface ξ and the receiving point x ;
-
R(x) is the distance between the origin O and the receiving point x .

The target surface S is discretized into $N_{s}$ surface elements and the receiving point x lies in the far-field of the integration surface S , the approximation r(x,ξ)≐R(x) holds. Consequently, the far-field scattered pressure can be further expressed as:

(2) $$p_{ff}^{s}(\overset{⌢}{x},{\hat{x}}^{inc})=\frac{1}{4\pi }\sum_{l=0}^{N_{s}}q_{l}({\hat{x}}^{inc})\int_{s_{0}}\left[ik\hat{x}\overset{⌢}{n}(\xi )+i]e^{-ik\hat{x}\delta (\xi )}dS(\xi \right),$$

where

-
$\hat{x}$ is the unit vector in the direction from the origin O to point x ;
-
$\hat{n}$ is the unit normal vector at that point ξ ;
-
$\delta \left(\xi \right)$ is the vector from point ξ to the origin O .

The scattered acoustic field matrix S is defined, with its elements denoted by $S_{mn}=p_{ff}^{s}({\overset{⌢}{x}}_{m},{\overset{⌢}{x}}_{n}^{inc})$ . The source density matrix Q is also defined, with its elements $Q_{ln}=q_{l}({\overset{⌢}{x}}_{n}^{inc})$ , resulting in:

(3) $$S=C^{ff}Q,$$

here,

-
S is a $m_{1}\times n_{1}$ -order matrix, where $m=1,2,\cdots ,m_{1}$ , with m denote the receiving angles, $m_{1}$ represents the total number of receiving angles. $n=1,2,\cdots ,n_{1}$ and n denote the incident angles, $n_{1}$ is the total number of incident angles;
-
$C^{ff}$ is a $m_{1}\times l_{1}$ -order matrix, where $l=1,2,\cdots ,l_{1}$ , l denotes the surface element index and $l_{1}$ is the total number of surface elements ( $l_{1}=N_{s}$ );
-
Q is a $l_{1}\times n_{1}$ -order matrix.

The acoustic scattering transfer matrix $C_{ml}^{ff}$ can be expressed as:

(4) $$C_{ml}^{ff}=\frac{1}{4\pi }\int_{s}\left[ik{\hat{x}}_{m}\overset{⌢}{n}(\xi )+i]e^{-ik{\hat{x}}_{m}\delta (\xi )}dS(\xi \right).$$

By rewriting the acoustic scattering matrix S and the sound source density function matrix Q in Equation (3) as column vectors $S_{ex}$ and $Q_{ex}$ , respectively. The corresponding transformation of $C_{ml}^{ff}$ is $C_{ex}$ , resulting in the system of equations

(5) $$S_{ex}=C_{ex}Q_{ex},$$

where the column vectors $S_{ex}$ and $Q_{ex}$ each contain $m_{1}n_{1}$ and $l_{1}n_{1}$ elements, and $C_{ex}$ is a $m_{1}n_{1}\times l_{1}n_{1}$ -order matrix.

In general, $m_{1}=n_{1}$ , according to the principle of acoustic reciprocity, the matrix S is symmetric. By eliminating the off-diagonal elements of the scattering pressure, we obtain a system of equations that only contains the diagonal elements of the acoustic scattering matrix. For the diagonal elements, the incident angle equals the receiving angle, which corresponds to the scattering sound pressure in a monostatic configuration. The resulting system after elimination consists entirely of monostatic pressures, which can be expressed as:

(6) $$S_{re}=C_{re}Q_{ex},$$

when the monostatic acoustic scattering matrix $S_{re}$ is known, i.e., the acoustic source density matrix $Q_{ex}$ in Equation (6) can be solved. Several sets of bistatic scattering matrices $S_{ad}$ are added to the monostatic scattering matrix $S_{re}$ . Meanwhile, the row vector corresponding to $S_{ad}$ is found in the acoustic scattering transfer matrix $C_{ex}$ , forming the matrix $C_{ad}$ , which is then appended to the matrix $C_{re}$ , resulting in the mixed acoustic scattering matrix $S_{s}$ and the mixed acoustic scattering transfer matrix $C_{s}$ . The expanded system of equations is as follows:

(7) $$S_{s}=C_{s}Q_{ex},$$

where,

(8) $$S_{s}=\left[\begin{array}{c} S_{re}\\ S_{ad} \end{array}\right],\;C_{s}=\left[\begin{array}{c} C_{re}\\ C_{ad} \end{array}\right],$$

The least squares method is used to approximate and solve Equation (7), yielding the acoustic source density matrix $Q_{ex}$ . The resulting column vector $Q_{ex}$ is then transformed into a $l_{1}\times n_{1}$ -order matrix Q , which is substituted into Equation (3) along with the acoustic scattering transfer matrix $C_{ml}^{ff}$ , producing the multi-static scattering sound pressure S .

### 2.2. Definition of Bistatic Reflection Coefficient

For a finite-sized coated plate, the scattered pressure at an incidence angle $\theta_{i}$ and reception angle $\theta_{r}$ is denoted as $p_{s}(\theta_{i},\theta_{r})$ . The corresponding reflection coefficient is defined as the ratio of the scattered pressure to the incident pressure in the free field. Thus, the reflection coefficient is given by:

(9) $$R(\theta_{i},\theta_{r})=\frac{p_{s}(\theta_{i},\theta_{r})}{p_{i}(\theta_{i})},$$

where $p_{i}(\theta_{i})$ is the incident plane-wave pressure in the absence of the plate.

In the monostatic case, where the transmitter and receiver are collocated $\theta_{r}=\theta_{i}$ , Equation (9) reduces to the classical monostatic reflection coefficient. In the bistatic case, with separate transmitter and receiver positions $\theta_{r}\neq \theta_{i}$ , the resulting reflection coefficient exhibits angular coupling characteristics, reflecting the directional dependence of scattering from the finite structure.

### 2.3. Numerical Implementation Process

To ensure clarity and reproducibility, the general approach and concrete steps taken for the numerical simulations and the fast prediction method are detailed as follows:

(1) The baseline numerical simulations are performed using the commercial software COMSOL Multiphysics 6.0, employing a multiphysics coupling approach to accurately capture the acoustic–structure interaction. Specifically, the “Pressure Acoustics, Frequency Domain” module is assigned to the fluid domain (water), while the “Solid Mechanics” module is applied to the structural domains (the rigid steel plate and the viscoelastic acoustic coating). These two physics interfaces are fully coupled at the acoustic–solid boundaries via the “Acoustic-Structure Boundary” node, ensuring the continuity of pressure and structural acceleration. The computational domain was truncated using a Perfectly Matched Layer (PML) to simulate an infinite water medium. The mesh size was strictly controlled, with the maximum element size set to λ/6 (one-sixth of the wavelength at the highest calculation frequency) to ensure convergence and accuracy. A plane wave with an amplitude of 1 Pa is applied in the external water domain along the xOz plane through the background pressure field module, and the incident angle is defined as 0° along the negative *z*-axis. The Helmholtz–Kirchhoff integral is solved using the external field calculation module with the full integral formulation.
(2) Acoustic Transfer Matrix Construction: The surface mesh model is numerically integrated to formulate the acoustic scattering transfer matrix (Equation (4)). This matrix depends solely on the target’s geometric shape and mesh.
(3) Sparse Data Sampling: This subset consists of monostatic (co-located transmitter–receiver) data and partially bistatic scattered pressure data (corresponding to the diagonal monostatic terms in Equation (6) and selected off-diagonal terms in the mixed system of Equation (7)).
(4) Source Density Inversion: The acoustic scattering transfer matrix is combined with the sparse subset of FEM data. A least-squares method is applied to approximate and solve the mixed system of equations (Equation (7)), yielding the equivalent acoustic source density matrix.
(5) Full-Angle Field Prediction: The retrieved acoustic source density matrix is substituted back into the scattering transformation framework alongside the transfer matrix to predict the complete multistatic scattering field across the full incident–receiving angular domain. Finally, by combining this predicted field with the reflection coefficient calculation formula (Equation (9)), the wide-angle bistatic reflection coefficients are rapidly extracted.

**Figure 2:**
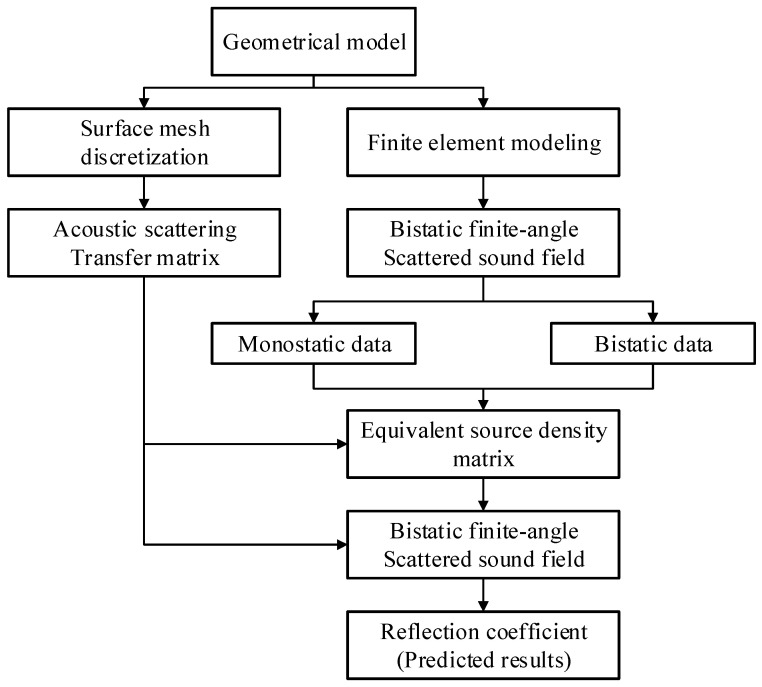
Computational Flowchart. The process highlights the combination of a sparse subset of FEM scattering data with the numerically integrated acoustic transfer matrix to retrieve the equivalent source density, ultimately outputting the full-angle bistatic reflection coefficients.

Figure 2 presents the computational flowchart of the proposed multistatic scattering field prediction method. The procedure consists of two main parts. The first part involves computing the full set of bistatic scattering field data within a limited angular domain using the finite element method (FEM). In the second part, the surface mesh model is numerically integrated to obtain the acoustic scattering transfer matrix. The transfer matrix is combined with monostatic and partially bistatic scattering field data obtained from FEM simulations. Using a least-squares inversion, the equivalent source density matrix is retrieved. This allows reconstruction of the complete scattering field within the selected angular range, reducing computational cost and improving efficiency.

## 3. Results and Discussion

The hardware and software environment used for simulations and data processing is detailed in Table 1.

### 3.1. Prediction of the Scattering Field for a Rigid Plate

**Figure 3:**
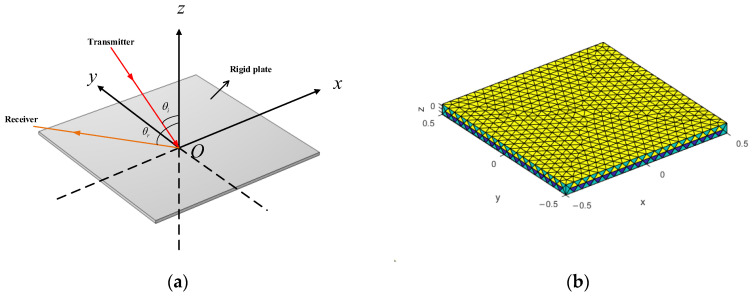
Schematic of the Rigid Plate Model: (**a**) Schematic of Acoustic Scattering Calculation; (**b**) Surface Mesh Model.

Figure 3 illustrates the geometry of the rigid plate model and its surface mesh discretization, with the mesh density set to one-sixth of the wavelength corresponding to the calculation frequency.

The rigid plate used in this study is made of steel, with a length and width of 1 m and a thickness of 0.008 m. A unit-amplitude plane wave is incident from the xOz plane. The ranges of incident and receiving angles are from −60° to 60° (with 0° defined along the negative *z*-axis) with a step of 1°. The calculation frequency spans from 1 kHz to 5 kHz with a step of 500 Hz.

**Figure 4:**
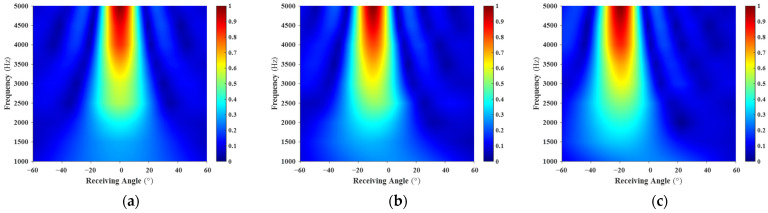
Angle–Frequency Spectrum of the Rigid Plate: (**a**) 0° Incidence; (**b**) 10° Incidence; (**c**) 20° Incidence. The spectra demonstrate that scattered energy is heavily concentrated along the specular reflection direction, with the main lobe narrowing progressively as frequency increases.

The multistatic scattering field of the rigid plate was obtained using the finite element method (FEM), and the corresponding angle–frequency spectrum is shown in Figure 4.

As shown in Figure 4, for the considered incident angles, the scattered energy is primarily concentrated near the specular reflection direction. At higher frequencies, the main reflection lobe becomes sharper, whereas in the low-frequency regime, the scattered energy exhibits a broader angular distribution. When the incident angle increases from 0° to 20°, the main reflection energy band shifts accordingly, with peak positions aligned with the corresponding incident angles, which is consistent with the characteristic specular reflection behavior of a finite rigid plate.

**Figure 5:**
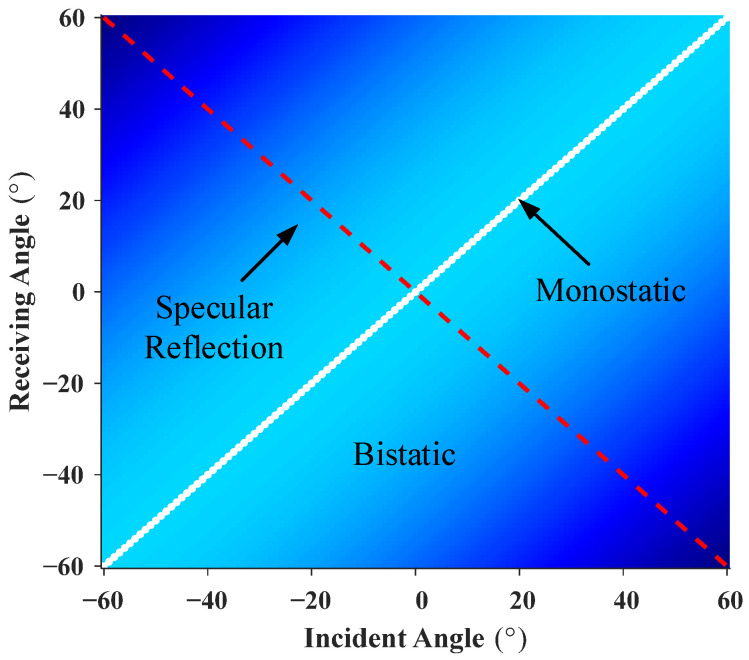
Range of scattered pressure data.

The monostatic (co-located transmitter–receiver) data were extracted from the FEM scattered pressure results, and partially bistatic data were sampled at 6° intervals, corresponding to the region below the monostatic diagonal in the incident–receiving angle map shown in Figure 5. This sampling strategy yields an input ratio of η = 2.31%, defined as the ratio of input elements to predicted elements. Based on this limited input, the equivalent source density was inverted to predict the full incident–receiving angle scattering field.

**Figure 6:**
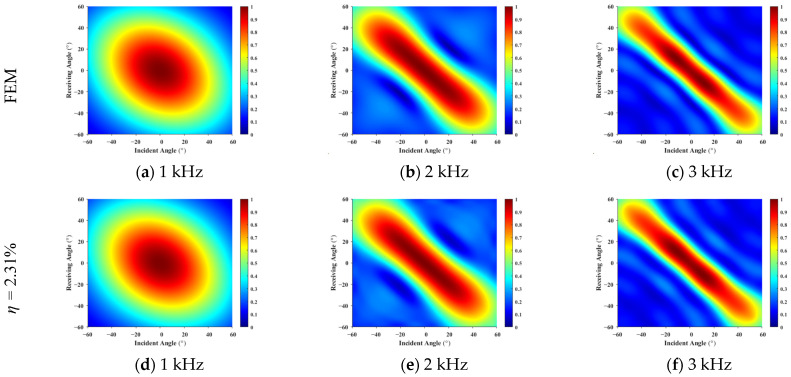
Scattered pressure versus incident–receiving angles for a rigid plate: (**a**–**c**) represent the finite element method results; (**d**–**f**) show the forecast results for 2.31%, respectively. The high consistency verifies the method’s accuracy in capturing interference fringes at varying frequencies.

Figure 6 compares the FEM results with the predicted results at different frequencies. The two sets of results show high consistency in terms of main-lobe positions, peak amplitudes, and angular coupling trends. In particular, the characteristic interference fringes observed at 3 kHz, which arise from finite-size scattering effects, are accurately reproduced by the proposed method, demonstrating its capability to capture high-frequency scattering details. Across the wide angular domain, the overall energy distribution of the predicted results agrees well with the FEM data, further validating the effectiveness of the method.

### 3.2. Prediction of Scattering Fields for Planar Structures with Acoustic Coating

**Figure 7:**
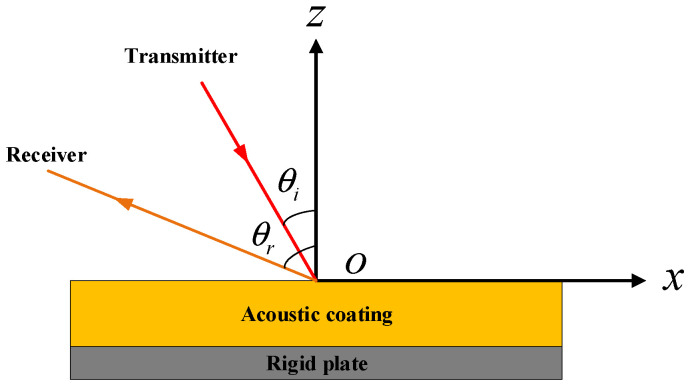
Schematic of the Planar Structure with Acoustic Coating.

As shown in Figure 7, a planar structure coated with an acoustic coating layer is considered. The model has a length and width of 1 m, a rigid plate thickness of 0.008 m, and a coating thickness of 0.05 m. The propagation medium is water, and a unit-amplitude plane wave is incident in the xOz plane. The incident and receiving angles range from −60° to 60°, with 0° defined along the negative *z*-axis and an angular step of 1°. The calculation frequency spans from 1 kHz to 5 kHz with a step of 500 Hz. Other parameters are kept identical to those of the rigid-plate case. Material properties of the coating and the plate are listed in Table 2 [32].

**Figure 8:**
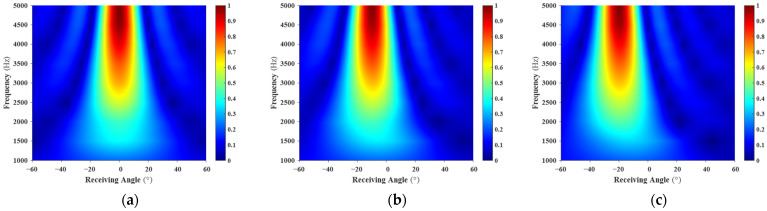
Angle–Frequency Spectrum of the Acoustically Coated Plate: (**a**) 0° Incidence; (**b**) 10° Incidence; (**c**) 20° Incidence. The spectra demonstrate that scattered energy is heavily concentrated along the specular reflection direction, with the main lobe narrowing progressively as frequency increases.

The multistatic scattering field of the planar structure with an acoustic coating was obtained using the FEM. The angle–frequency spectrum of the FEM results is shown in Figure 8.

As shown in Figure 8, for all three incident angles, the scattered energy is primarily concentrated near the specular reflection direction. Compared with the rigid plate, however, the scattering exhibits a broader angular distribution. When the incident angle increases from 0° to 20°, the main reflection energy band shifts noticeably, with the peak positions corresponding to the incident directions.

**Figure 9:**
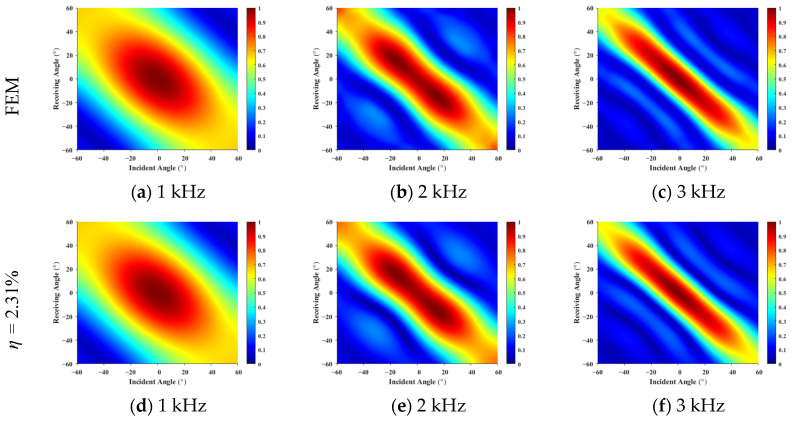
Scattered pressure versus incident–receiving angles for the acoustically coated plate: (**a**–**c**) represent the finite element method results; (**d**–**f**) show the forecast results for 2.31%, respectively. The high consistency verifies the method’s accuracy in capturing interference fringes at varying frequencies.

Figure 9 compares the scattered pressure amplitude distributions obtained from the FEM simulations and the predicted results using an input ratio of η = 2.31% at frequencies of 1 kHz, 2 kHz, and 3 kHz.

The results in Figure 9 show that at 1 kHz, the scattering pattern is characterized by a broad main lobe with a relatively uniform angular variation, and the predicted results closely match the FEM data in both overall shape and angular energy distribution. As the frequency increases, the predicted results accurately reproduce the interference fringes observed in the FEM results, including their tilt, spacing, and angular modulation. Over the full angular range from −60° to 60°, the predicted fields preserve the dominant scattering features of the coated plate, demonstrating the effectiveness of the proposed method even with a very limited input dataset.

**Figure 10:**
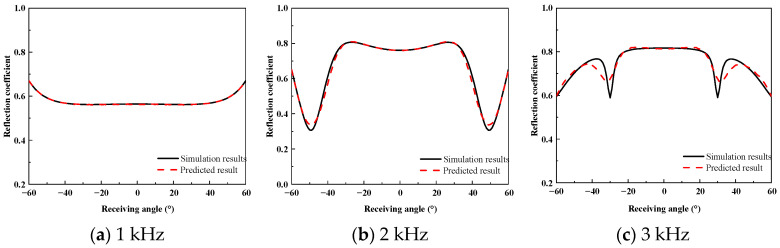
High-Frequency Reflection Coefficient at 0° Incidence.

**Figure 11:**
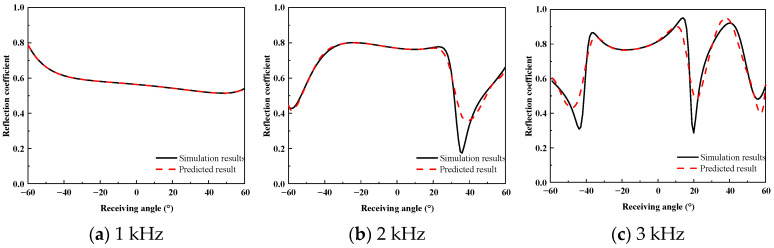
High-Frequency Reflection Coefficient at 10° Incidence.

**Figure 12:**
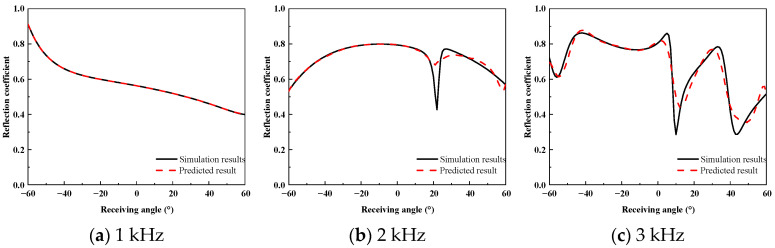
High-Frequency Reflection Coefficient at 20° Incidence.

To assess the prediction accuracy of the proposed method for the wide-angle reflection coefficients of the acoustically coated plate, a comparative analysis was performed between the FEM simulation results and the predicted results. Figure 10, Figure 11 and Figure 12 present the reflection coefficient curves corresponding to incident angles of 0°, 10°, and 20°, respectively.

As shown in Figure 10, Figure 11 and Figure 12, the reflection coefficients vary smoothly at low frequencies, and the predicted curves show close agreement with the FEM results. In the 2–3 kHz frequency band, dispersion effects in the coating lead to pronounced multi-peak features, which are accurately reproduced by the proposed method in terms of both peak locations and amplitudes. Similar complex energy dissipation and modal behaviors have been thoroughly documented in the literature; for instance, Gao et al. [20] and Ranjbar et al. [22] demonstrated that the introduction of specific structures and viscoelastic damping in acoustic coatings heavily modulates the frequency-dependent reflection characteristics. The ability of the proposed equivalent source density inversion to accurately reconstruct these localized physical phenomena—without relying on heavy full-wave sweeps—highlights its superiority over standard ray-tracing or physical acoustics approximations, which frequently fail to capture the interfacial dissipation and elasticity of high-loss coatings.

### 3.3. Analysis of Low-Frequency Characteristics

**Figure 13:**
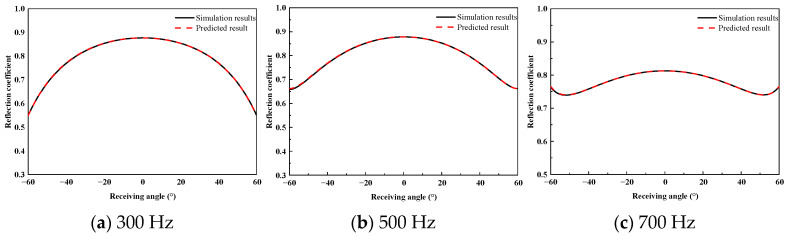
Low-Frequency Reflection Coefficient at 0° Incidence.

**Figure 14:**
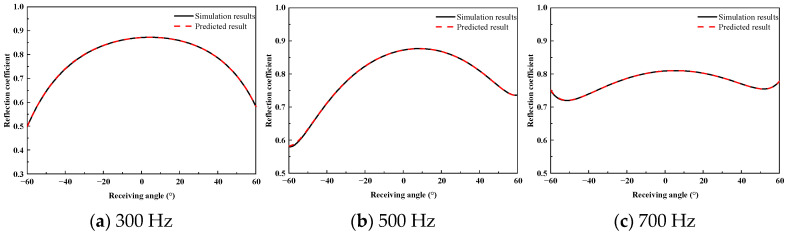
Low-Frequency Reflection Coefficient at 10° Incidence.

**Figure 15:**
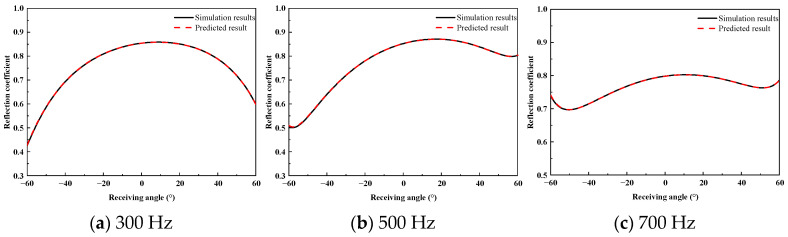
Low-Frequency Reflection Coefficient at 20° Incidence.

To verify the method’s applicability in the low-frequency regime, the prediction capabilities were extended to the frequency band of 100 Hz to 1 kHz with a step of 100 Hz. Figure 13, Figure 14 and Figure 15 present the comparison between the FEM simulation results and the predicted reflection coefficients at representative frequencies for incident angles of 0°, 10°, and 20°, respectively.

As can be seen from the figures, the reflection coefficient curves vary smoothly and do not exhibit the interference fringes observed in the high-frequency response. This characteristic arises because, within this frequency range, the acoustic wavelength is larger than the characteristic dimensions of the plate, the influence of internal resonances in the coating is weakened, and the overall diffraction effects of the finite-sized plate structure dominate. The comparison shows that the predicted results are in excellent agreement with the finite element benchmarks for all considered incident angles.

### 3.4. Error Analysis and Discussion

**Figure 16:**
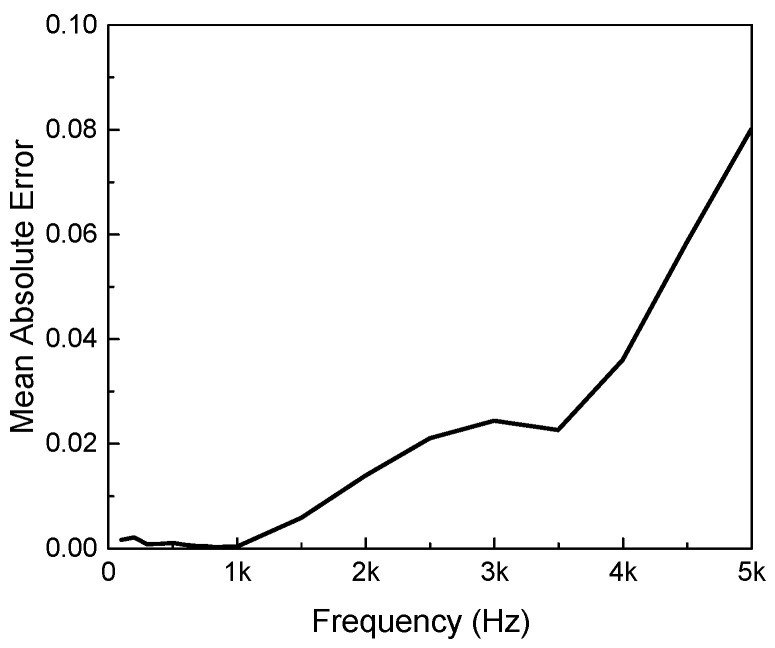
Mean Absolute Error (MAE) of the Reflection Coefficients. In the low-frequency regime, the error remains extremely low (MAE < 0.03), while at higher frequencies, the error shows a slight and acceptable increase as the equivalent source density function becomes more complex.

To quantitatively evaluate the accuracy of the proposed method across the entire investigation bandwidth. Figure 16 shows the frequency-dependent trend of the mean absolute error (MAE) between the reflection coefficients predicted by the proposed method and the finite element results in the 100 Hz–5 kHz frequency range.

As shown in the figure, the error evolution can be categorized into two distinct regimes. Within the 100 Hz–3.5 kHz frequency range, the MAE of the reflection coefficients remains below 0.03, indicating that the predicted results are stable and accurate. The MAE of the predicted reflection coefficients tends to rise with frequency. This is because at lower frequencies, the source density function is relatively simple, and a combination of monostatic data with a limited amount of bistatic data is sufficient for accurate estimation. However, the source density function becomes increasingly complex with frequency, requiring more bistatic data for precise estimation [31].

Nevertheless, the MAE remains within an acceptable range across the entire frequency band. For the simulation of reflection coefficients of acoustic coating, the proposed method can significantly reduce computational cost and achieve high accuracy at low and medium frequencies. For experimental measurements of reflection coefficients, the method overcomes the difficulty of obtaining full-angle reflection coefficients, enabling the prediction of full-angle reflection coefficients using only limited experimental measurement data.

### 3.5. Computational Efficiency Comparison

To explicitly demonstrate the efficiency of the proposed fast prediction method, a direct comparison of the computational time required for a traditional FEM full-angle sweep versus the proposed approach was conducted in Table 3. For the calculation of 18 frequency points across the full incident–receiving angle combinations, the traditional FEM full-wave sweep requires a total calculation time of 14,328 s. In contrast, the proposed fast prediction method—utilizing only a sparse subset of input data—completes the full-angle prediction for the same operational conditions in just 3762 s. This represents a substantial reduction in computational burden, proving the method’s high efficiency.

In the proposed prediction framework, the estimation of the acoustic source density function via the least squares method constitutes the primary computational step. This algorithm seeks the optimal equivalent source density by minimizing the sum of squared errors. Increasing the proportion of known bistatic scattering data can accelerate the calculation speed. A richer input data set provides more boundary constraints, enabling the least squares solver to converge more rapidly on a source density function that satisfies the accuracy requirements [30].

## 4. Conclusions

This work addresses the problem of predicting multistatic scattering fields of planar structures with acoustic coatings under wide-angle conditions and proposes a fast reflection coefficient prediction method based on a limited set of scattered pressure data. The method represents the scattered field as the product of an acoustic scattering transfer matrix, which depends solely on the geometric shape, and the equivalent source density. By inverting the source density using monostatic and partially bistatic scattered pressure measurements, the full-angle reflection coefficients can be rapidly obtained. Based on comparisons with FEM results, the following conclusions are drawn:

(1) Simulation results for both the rigid plate and the acoustically coated plate demonstrate that the proposed method can accurately reproduce the specular reflection main lobe, the wide-angle decay trend, and high-frequency interference patterns. The predicted results show a high degree of agreement with the FEM data.
(2) In the 100 Hz–5 kHz frequency range, the proposed method maintains excellent prediction performance for the acoustically coated plate, even in the presence of material loss.
(3) The method requires only monostatic and partially bistatic scattering data to reconstruct the full-angle scattering field, effectively avoiding the computational burden associated with full angular sweeps and potentially enabling prediction of the scattering field using limited experimental measurement data.

Although this study lays a theoretical and numerical foundation for the fast prediction of reflection coefficients, several directions deserve further exploration to enhance its practical value. First, experimental validation in a laboratory water tank or open-water environment is planned to further examine the robustness of the method against measurement noise and environmental uncertainties. Second, the proposed fast prediction framework will be combined with the Planar Element Method (PEM). By using the reconstructed wide-angle reflection coefficients as local boundary conditions, this hybrid approach can efficiently compute the Target Strength (TS) of complex underwater structures coated with acoustic claddings. Such an extension is expected to provide a highly efficient tool for the acoustic design and stealth performance evaluation of large-scale targets in multistatic sonar systems. Furthermore, the nonlinear behavior of the reflection coefficient in the 3–5 kHz range offers significant practical value for solving detection problems. The pronounced interference fringes and angular coupling effects observed in this frequency band, which are induced by the internal resonance of the viscoelastic coating, can serve as identifiable ‘acoustic fingerprints’ of the target. In practical multistatic sonar applications, capturing these unique scattering signatures allows for the differentiation of coated targets from rigid objects or environmental clutter, thereby providing a physical basis for enhancing target classification and recognition performance in complex underwater environments.

## Figures and Tables

**Table 1:** Hardware and Software Specifications.

Item	Specification
CPU	AMD Ryzen 5 3600X 6-Core Processor 3.79 GHz
Memory (RAM)	32.0 GB
GPU	NVIDIA GeForce GT 710 (2 GB)
Simulation Software	COMSOL Multiphysics 6.0
Data Processing	MATLAB R2023b

**Table 2:** Material Properties.

Material	Density [kg·m^−3^]	Young’s Modulus [Pa]	Poisson Ratio	Sound Velocity [m·s^−1^]	Thickness [mm]
Rubber	1090	3 × 10^7^ (1 + 0.249i)	0.49	/	50
Steel	7800	2.13 × 1011	0.3	/	8
Water	1000	/	/	1500	/

**Table 3:** Full Frequency Band Computational Time Comparison.

Method	Number of Mesh Elements	Time (s)
FEM	720,700	14,328
Fast Prediction Method (η = 2.31%)	2238	3762

## Data Availability

All evaluated data are presented in this paper in graphical form. Original measurement data of this study are available upon request from the corresponding author.
